# Fecal Short-Chain Fatty Acid Variations by Breastfeeding Status in Infants at 4 Months: Differences in Relative versus Absolute Concentrations

**DOI:** 10.3389/fnut.2017.00011

**Published:** 2017-04-10

**Authors:** Sarah L. Bridgman, Meghan B. Azad, Catherine J. Field, Andrea M. Haqq, Allan B. Becker, Piushkumar J. Mandhane, Padmaja Subbarao, Stuart E. Turvey, Malcolm R. Sears, James A. Scott, David S. Wishart, Anita L. Kozyrskyj, The CHILD Study Investigators, M. R. Sears

**Affiliations:** ^1^Department of Pediatrics, University of Alberta, Edmonton, AB, Canada; ^2^Department of Pediatrics and Child Health, Children’s Hospital Research Institute of Manitoba, University of Manitoba, Winnipeg, MB, Canada; ^3^Department of Agricultural, Food and Nutritional Science, University of Alberta, Edmonton, AB, Canada; ^4^Department of Pediatrics, University of Toronto, Toronto, ON, Canada; ^5^Department of Pediatrics, University of British Columbia, Vancouver, BC, Canada; ^6^St Joseph’s Healthcare and Department of Medicine, Firestone Institute for Respiratory Health, McMaster University, Hamilton, ON, Canada; ^7^Dalla Lana School of Public Health, University of Toronto, Toronto, ON, Canada; ^8^Department of Biological Sciences, University of Alberta, Edmonton, AB, Canada; ^9^Canadian Healthy Infant Longitudinal Development Study, Hamilton, ON, Canada

**Keywords:** short-chain fatty acids, lactate, succinate, breastfeeding, infants, gut microbiota

## Abstract

Our gut microbiota provide a number of important functions, one of which is the metabolism of dietary fiber and other macronutrients that are undigested by the host. The main products of this fermentation process are short-chain fatty acids (SCFAs) and other intermediate metabolites including lactate and succinate. Production of these metabolites is dependent on diet and gut microbiota composition. There is increasing evidence for the role of SCFAs in host physiology and metabolic processes as well as chronic inflammatory conditions such as allergic disease and obesity. We aimed to investigate differences in fecal SCFAs and intermediate metabolites in 163 infants at 3–5 months of age according to breastfeeding status. Compared to no exposure to human milk at time of fecal sample collection, exclusive breastfeeding was associated with lower absolute concentrations of total SCFAs, acetate, butyrate, propionate, valerate, isobutyrate, and isovalerate, yet higher concentrations of lactate. Further, the relative proportion of acetate was higher with exclusive breastfeeding. Compared to non-breastfed infants, those exclusively breastfed were four times more likely (aOR 4.50, 95% CI 1.58–12.82) to have a higher proportion of acetate relative to other SCFAs in their gut. This association was independent of birth mode, intrapartum antibiotics, infant sex, age, recruitment site, and maternal BMI or socioeconomic status. Our study confirms that breastfeeding strongly influences the composition of fecal microbial metabolites in infancy.

## Introduction

Gut microbiota have coevolved over millennia in a largely symbiotic relationship with the host. For the majority of time, human milk has been the sole source of nutrition for infants, providing essential nutrients for infant growth, as well as bioactive components to stimulate the gut microbiome. Both breastfeeding and gut microbial composition have been associated with a number of health outcomes during infancy and later life (1). In addition to providing a wide range of pathogen exclusion and immune and biosynthesis functions (2), a major role of gut microbiota is the metabolism of dietary fiber and other complex macronutrients that escape digestion in the small intestine.

The main products of nutrient breakdown by microbes are short-chain fatty acids (SCFAs), the predominant ones being acetate, butyrate, and propionate and to a lesser degree branched-chain fatty acids (BCFAs), valerate, isobutyrate, and isovalerate (3). Less well studied are lactate and succinate, intermediate metabolites in the microbial production of SCFAs (4). In adults, the majority of the SCFAs are rapidly absorbed or used as an energy source by colonocytes (5). There is increasing evidence that gut microbial metabolites have wider systemic effects in the host through their action as signaling molecules and involvement in regulators of gene expression (6, 7). SCFAs have been linked to appetite suppression by activating free fatty acid receptors in the intestine and increasing circulating anorectic gut hormones (3). They have also been shown to play an important role in the activation and differentiation of immune cells and have been implicated in inflammatory and allergic disease (3, 8, 9).

The quantity and relative proportion of metabolites produced in the colon of adults has been closely linked to diet, as well as microbiota composition, diversity, and activity (10). Infancy represents a rapid period of gut microbial development, which is shaped by early-life events such as birth mode, antibiotic administration, and importantly, infant feeding (11–13). Human milk provides optimal nutrition for infants in the first 6 months of life and contains significant amounts of carbohydrates that escape digestion in the small intestine, identified as human milk oligosaccharides (HMOs), which are the preferred substrates for certain gut microbiota in the production of SCFAs (14). Studies have shown that infants who are exclusively breastfed have lower microbial diversity, with a predominance in Proteobacteria and Actinobacteria (most notably bifidobacteria), whereas formula-fed infants tend to have a more diverse microbiota with increased abundance of Clostridia and *Bacteroides* species (15–17).

Gut microbiota dysbiosis and subsequent changes to metabolite profiles may be particularly important in infancy, which presents a critical window of opportunity in programming of future metabolic and immune health. Understanding how diet in early life can shape gut microbiota-associated metabolites is therefore of interest. While studies have previously reported differences in fecal SCFAs by infant diet (18–24), many had a small sample size, and few studies have reported on differences in relative proportions of SCFAs or intermediate metabolites. In addition, limited studies to date have assessed the impact of other birth factors, implicated in early microbiota development, on these associations (21).

We aimed to investigate differences in fecal SCFAs and intermediate metabolites in infants at 3–5 months of age according to breastfeeding. Specifically, we investigated whether fecal total and individual SCFAs, lactate, and succinate (measured as both absolute concentrations and relative proportions) differed according to breastfeeding status at the time of metabolite measurement and duration of exclusive breastfeeding. A secondary aim was to investigate whether these associations were independent of birth mode and intrapartum antibiotics use, as well as other early-life factors.

## Materials and Methods

### Study Design and Covariates

The study included an unselected subset of 163 infants from the Canadian Healthy Infant Longitudinal Development national population-based birth cohort (25) (www.canadianchildstudy.ca) whose mothers were enrolled between November 2009 and February 2012 and who had fecal samples available for analysis collected between 3 and 5 months of age.

Mothers reported infant feeding practices using standardized questionnaires administered at 3, 6, and 12 months postpartum. Questionnaires asked mothers to record if they had ever breastfed their child, if they were currently breastfeeding their child, and the age of the child when breastfeeding ceased, if applicable. Mothers were also asked questions regarding formula feeding initiation and cessation. From these questionnaires, variables on feeding habits between birth and fecal sample collection were calculated. These included whether infants had ever been breastfed, duration of exclusive breastfeeding (never, <3 months, and ≥3 months), and breastfeeding status at the time of fecal sample collection (exclusively breastfed, partially breastfed, and not breastfed).

Mode of birth [classified as vaginal, elective, or emergency cesarean section (CS)] and maternal intrapartum antibiotic prophylaxis (IAP) were extracted from hospital records. This was used to create a four-category variable for birth mode/IAP exposure: vaginal birth (no IAP), vaginal birth IAP, elective cesarean, and emergency cesarean, which we have previously reported on in association with infant gut microbiota (11). Information on other covariates including maternal age, ethnicity and education, infant sex, gestational age, and birth weight were obtained from hospital records or through standardized questionnaires completed by mothers. Maternal weight status [body mass index, weight (kilograms)/height (square meter)] was calculated from height and weight data taken from hospital records or measured at the 1-year postpartum clinic visit. There was a small amount of missing data for some variables (see Table 1).

**Table 1:** **Study characteristics**.

Characteristic	
**Mean (SD)**	
Maternal age (years) (*n* = 162)	32 (4.7)
Gestational age (weeks) (*n* = 155)	38.9 (1.5)
Birth weight (g) (*n* = 158)	3,462 (503)
Age at stool sample collection (months), *n* (%)	3.65 (0.47)
**City of birth, *n* (%)**	
Edmonton	52 (32)
Vancouver	95 (58)
Winnipeg	16 (10)
**Infant sex, *n* (%)**	
Male	90 (55)
Female	73 (45)
**Maternal ethnicity, *n* (%) (*n* = 160)**	
Caucasian	119 (74)
Asian	24 (15)
Other	17 (11)
**Birth mode, *n* (%) (***n*** = 158)**	
Vaginal—no IAP	78 (49)
Vaginal—IAP	31 (20)
Cesarean section—emergency	29 (18)
Cesarean section—elective	20 (13)
**Breastfeeding status at fecal sample collection, *n* (%) (*n* = 158)**	
None	44 (28)
Partial	66 (42)
Exclusive	48 (30)
**Ever breastfed, *n* (%)**	
No	17 (10)
Yes	146 (90)
**Exclusive breastfeeding duration, *n* (%)**	
Never	51 (31)
<3 months	54 (33)
≥3 months	58 (36)

*N = 163 unless otherwise specified*.

*IAP, intrapartum antibiotic prophylaxis*.

### Sample Collection, Preparation, and Nuclear Magnetic Resonance (NMR) Analysis

Samples were analyzed using NMR spectroscopy, which allows for the simultaneous measurement of a wide range of metabolites in a sample and has been successfully applied to metabolite measurement in fecal samples (26). Fecal samples (fresh or refrigerated for a short period) were collected using a standard protocol at a home visit at a mean age of 3.7 months (SD 0.47). Samples were refrigerated during transport and stored at −80°C until analysis.

Approximately 100 mg of fecal sample was homogenized and quickly transferred to an Eppendorf tube. One milliliter of ice cold water was added to the fecal powder and vortexed vigorously for 5 min followed by sonication at 4°C for 20 min. The samples were further subjected to vortex shaking at 250 rpm for 20 min. The fecal water extract thus obtained was centrifuged at 15,000 × *g* for 1 h at 4°C, and the supernatant was carefully aspirated into a fresh Eppendorf tube. This supernatant was centrifuged again at 15,000 × *g* for 1 h at 4°C to precipitate any particulate fecal matter (that might have been introduced during the first separation), and the clear extract was transferred into a clean Eppendorf tube. The resultant fecal water was stored at −20°C until further analysis.

A 570 μL aliquot of fecal water was placed in a 1.5-mL Eppendorf tube followed by the addition of 70 μL of D_2_O and 60 μL of standard buffer solution [585 mM NaHPO_4_ (pH 7.0), 11.667 mM disodium-2,2-dimethyl-2-silapentane-5-sulfonate (DSS), and 0.47% NaN_3_ in H_2_O]. The samples (700 μL) were then transferred to a regular NMR tube for subsequent NMR spectral analysis.

All ^1^H-NMR spectra were collected on a Varian 500 MHz Inova spectrometer equipped with a 5-mm HCN Z-gradient pulsed-field gradient cyrogenic probe. ^1^H-NMR spectra were acquired at 25°C using the first transient of the Varian tnnoesy pulse sequence, which was chosen for its high degree of selective water suppression and quantitative accuracy of resonances around the solvent. Water suppression pulses were calibrated to achieve a bandwidth of 80 G. Spectra were collected with 128 transient and 8 steady-state scans using a 4-s acquisition time (48,000 complex points) and a 1-s recycle delay.

Before spectral analysis, all free induction decays were zero-filled to 64,000 data points and line broadened 0.5 Hz. The methyl singlet produced by a known quantity of DSS was used as an internal standard for chemical shift referencing (set to 0 ppm) and for quantification. All ^1^H-NMR spectra were processed and analyzed using the Chenomx NMR Suite Professional software package version 8.1 (Chenomx Inc., Edmonton, AB, Canada). The Chenomx NMR Suite software allows for qualitative and quantitative analysis of an NMR spectrum by manually fitting spectral signatures from an internal database to the spectrum. Typically 90% of visible peaks were assigned to a compound, and more than 90% of the spectral area could be routinely fit using the Chenomx spectral analysis software. Most of the visible peaks were annotated with a compound name.

### Statistical Analysis

Fecal metabolites were analyzed as absolute concentrations (micromoles per gram of feces) and as relative proportions (%) of total SCFAs/BCFAs (labelled as SCFAs for short, and taken as the sum of acetate, butyrate, propionate, valerate, isobutyrate, and isovalerate). Median concentrations of total SCFAs (acetate, butyrate, propionate, valerate, isobutyrate, and isovalerate) and individual SCFAs as well as lactate and succinate were compared according to breastfeeding duration and breastfeeding at time of fecal sample collection using Kruskal–Wallis test (non-parametric ANOVA, with Bonferroni posttest for multiple comparison). Median relative proportions of SCFAs were also compared using Kruskal–Wallis test (with Bonferroni posttest).

Given the skewed nature of the data, fecal metabolites were categorized into a binary variable (high and low) using the median as a cut point, and associations with breastfeeding at stool collection were tested using logistic regression. Models were adjusted for birth mode and IAP exposure, age of stool sample collection as well as maternal and infant factors found to be associated with breastfeeding in our study (infant sex, city of birth, maternal education and BMI). All analysis was conducted using IBM SPSS (version 24). Statistical significance was considered when *P* ≤ 0.05.

## Results

Fecal samples were collected from 163 infants between the ages of 3–5 months (mean age 3.65 ± 0.47 months). Subject characteristics of our sample are shown in Table 1. At fecal sample collection, 30% were exclusively breastfed and 42% of infants were partially breastfed. Twenty-two (14.2%) infants had started solid foods. The majority of infants (69%) were born vaginally, while almost a third of infants (31%) were born by CS. Twenty-eight percent of vaginally delivered infants received IAP, and all cesarean infants received IAP as is standard practice in Canada.

Breastfeeding exclusivity at fecal sample collection differed by city of birth (Table 2). Males were less likely to be exclusively breastfed than females (25 versus 37%), as were infants born to overweight or obese mothers and mothers with a lower level of education. Breastfeeding also differed according to birth mode, with exclusive breastfeeding at the time of fecal collection being highest in vaginally delivered infants not exposed to IAP and lowest in elective CS infants (34 and 15%, respectively), although this difference was not statistically significant (chi-square *P* = 0.16).

**Table 2:** **Associations between breastfeeding and subject characteristics**.

Characteristic	Breastfeeding status, *N* (%)			
	None	Partial	Exclusive	*P* value
**City of birth**				
Edmonton	19 (37)	25 (49)	7 (14)	0.002
Vancouver	18 (20)	35 (38)	39 (42)	
Winnipeg	7 (47)	6 (40)	2 (13)	
**Infant sex**				
Male	22 (25)	44 (50)	22 (25)	0.06
Female	22 (31)	22 (31)	26 (37)	
**Maternal ethnicity (***n*** = 155)**				
Caucasian	31 (27)	50 (44)	33 (29)	0.96
Asian	6 (25)	10 (42)	8 (33)	
Other	5 (29)	6 (35)	6 (35)	
**Maternal education (***n*** = 155)**				
Less than University	29 (46)	23 (37)	11 (18)	<0.001
University or higher	14 (15)	42 (46)	36 (39)	
**Maternal BMI (kg/m^2^) (***n*** = 155)**				
Normal (<25)	20 (22)	35 (39)	36 (40)	0.02
Overweight or obese (≥25)	23 (36)	29 (45)	12 (19)	
**Birth weight (***n*** = 153)**				
≤4 kg	38 (29)	54 (41)	41 (31)	0.45
>4 kg	5 (25)	11 (55)	4 (20)	
**Gestational age (***n*** = 150)**				
Early term (<38 weeks)	4 (20)	9 (45)	7 (35)	0.78
Term (38–40)	33 (30)	45 (41)	32 (29)	
Late term (41+ weeks)	6 (30)	10 (50)	4 (20)	
**Birth mode (***n*** = 153)**				
Vaginal—no IAP	24 (32)	26 (34)	26 (34)	0.34
Vaginal—IAP	5 (17)	16 (55)	8 (28)	
Cesarean section—emergency	8 (29)	11 (39)	9 (32)	
Cesarean section—elective	6 (30)	11 (55)	3 (15)	

*Breastfeeding status at fecal sample collection. N = 158 unless otherwise specified. P value Pearson chi-square test*.

*IAP, intrapartum antibiotic prophylaxis*.

### Absolute Concentrations of SCFAs and Intermediate Metabolites and Breastfeeding Status

Concentrations (micromoles per gram of feces) of SCFAs, lactate, and succinate in all infants are presented in Table 3. Total SCFA concentration was 142.0 μmol/g with the most abundant SCFA being acetate followed by propionate and butyrate.

**Table 3:** **Median concentrations of short-chain fatty acid (SCFA) and intermediate metabolites according to breastfeeding status and duration**.

Metabolite (μmol/g) [interquartile range (IQR)]	All infants (*n* = 163)	Breastfeeding status^a^ (*n* = 158)			Ever breastfed (*n* = 163)		Exclusive breastfeeding duration (*n* = 163)		
		None (*n* = 44)	Partial (*n* = 66)	Exclusive (*n* = 48)	No (*n* = 17)	Yes (*n* = 146)	Never (*n* = 51)	<3 months (*n* = 54)	≥3 months (*n* = 58)
Total SCFA^b^	142.0 (101.6–203.8)	177.8 (125.7–241.6)	161.4 (126.1–233.6)	99.4*** (63.8–130.5)	185.0 (135.1–239.7)	138.9 (96.3–199.3)	176.0 (134.1–239.9)	173.6 (122.5–235.5)	96.9*** (60.4–133.4)
Acetate	116.5 (74.9–165.6)	135.6 (86.2–190.5)	128.8 (81.0–179.3)	79.4*** (52.7–121.3)	132.8 (109.4–193.9)	115.0 (73.2–164.7)	146.5 (97.2–200.8)	126.5 (87.0–185.6)	75.9*** (50.5–123.6)
Butyrate	6.6 (1.9–12.5)	11.7 (5.8–18.0)	7.1* (3.7–11.6)	1.6*** (0.44–7.2)	11.5 (6.1–15.8)	6.1* (1.7–12.0)	9.2 (4.8–13.9)	7.8 (3.7–16.3)	1.8*** (0.47–7.2)
Propionate	14.5 (4.6–28.4)	19.7 (13.7–32.7)	19.6 (9.6–34.1)	4.3*** (1.7–11.1)	22.1 (17.4–34.8)	12.7* (4.3–27.2)	22.5 (13.5–34.9)	17.2 (10.9–35.3)	4.7*** (1.9–13.1)
Valerate	1.2 (0.42–2.3)	1.9 (1.2–2.8)	1.3 (0.60–2.4)	0.39*** (0.15–1.2)	1.7 (0.72–2.8)	1.2 (0.41–2.3)	1.7 (0.66–2.8)	1.6 (0.81–2.8)	0.41*** (0.19–1.3)
Isobutyrate	0.67 (0.22–1.7)	1.3 (0.69–2.2)	0.59 (0.26–2.0)	0.20*** (0.07–0.72)	1.6 (0.77–3.3)	0.57** (0.20–1.7)	1.2 (0.50–1.2)	0.83 (0.40–2.2)	0.20*** (0.08–0.69)
Isovalerate	1.0 (0.34–2.4)	1.9 (1.2–3.5)	1.3* (0.43–2.8)	0.33*** (0.09–0.91)	3.0 (1.8–3.9)	0.92*** (0.30–2.2)	1.6 (0.79–3.1)	1.6 (0.80–3.1)	0.36*** (0.10–0.95)
Lactate	3.7 (1.5–18.5)	2.3 (1.3–3.2)	4.7* (1.5–12.4)	7.2*** (2.2–32.7)	2.4 (1.4–2.7)	4.7 (1.6–20.2)	3.5 (1.6–15.7)	2.5 (1.2–8.2)	6.6 (2.2–29.9)
Succinate	10.4 (3.1–29.6)	4.3 (1.6–17.7)	21.8*** (4.2–49.7)	8.4 (3.4–30.6)	3.3 (1.9–18.7)	11.9 (3.4–34.5)	7.9 (2.7–24.5)	11.3 (3.5–31.8)	10.6 (3.2–37.1)

*Values are presented as median and IQR in micromoles per gram of feces. Comparisons by non-parametric Mann–Whitney *U* test (two groups) or Kruskal–Wallis test (three groups, with Bonferroni posttest for multiple comparison) versus none or never breastfeeding category*.

***P < 0.001.

**P < 0.01.

*P < 0.05.

*^a^Breastfeeding status at stool sample collection*.

*^b^Total SCFAs represent the sum of acetate, butyrate, propionate, valerate, isobutyrate, and isovalerate*.

In univariate analysis, infants who had ever been breastfed had lower concentrations of total SCFAs, acetate, butyrate, propionate, valerate, isobutyrate, and isovalerate and higher concentrations of lactate and succinate than those who had never been breastfed (Table 3). Compared to those not being breastfed at time of fecal metabolite profiling, those who were exclusively breastfed had significantly lower total SCFAs concentrations and lower concentrations of all individual SCFAs (Table 3). Lactate was significantly higher in those exclusively breastfed versus those not breastfed (7.2 versus 2.3 μmol/g; *P* < 0.001). Those who were partially breastfed had similar concentrations of total SCFAs, acetate, and propionate compared to those not breastfed at the time of fecal metabolite profiling or concentrations falling midway between those not breastfed and those exclusively breastfed (seen for butyrate, valerate, isobutyrate, isovalerate, and lactate). Similar differences were observed according to duration of exclusive breastfeeding with those breastfed for ≥3 months having significantly lower concentrations of SCFAs than infants who had never been breastfed (Table 3). Similar results were observed when data were restricted to infants who had not started solid foods (Table S1 in Supplementary Material) and vaginally delivered infants without exposure to IAP (Table S3 in Supplementary Material).

### Relative Proportions of SCFAs and Breastfeeding

**Figure 1:**
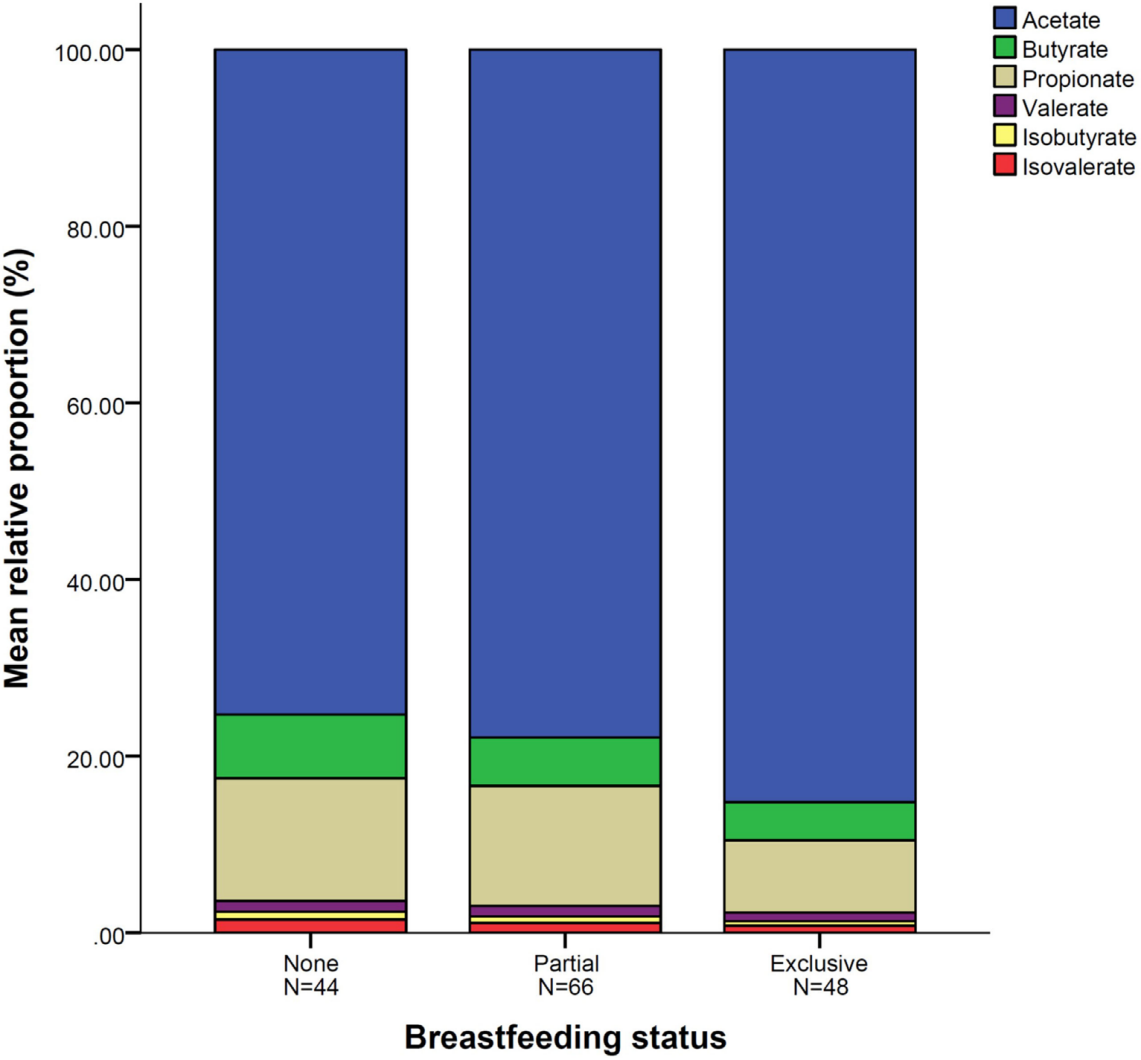
**Mean relative proportion of short-chain fatty acid according to breastfeeding status**. *N* = 158. Breastfeeding status at fecal sample collection.

In all infants, acetate made up the largest proportion of the SCFAs (80%) followed by propionate (10%) and butyrate (5%) with isovalerate being the least abundant (Table 4). In univariate analysis, infants exclusively breastfed at time of fecal metabolite profiling had significantly higher relative proportions of acetate and lower proportions of butyrate, propionate, isobutyrate, and isovalerate compared to those not breastfed (Table 4; Figure 1). Similar results were seen for those exclusively breastfed ≥3 months versus those never exclusively breastfed (Table 4). Compared to infants who had never been breastfed, breastfed infants also tended to have higher proportions of acetate and lower proportions of butyrate, propionate, isobutyrate, and isovalerate although differences were only significant for the latter two metabolites. Similar trends were observed when data were restricted to infants who had not started solid foods (Table S2 in Supplementary Material) and infants vaginally delivered without exposure to IAP (Table S4 in Supplementary Material), although loss in statistical significance was observed in some instances.

**Table 4:** **Relative proportions of total short-chain fatty acid according to breastfeeding status and duration**.

Metabolite% [interquartile range (IQR)]	All infants (*n* = 163)	Breastfeeding status^a^ (*n* = 158)			Ever breastfed (*n* = 163)		Exclusive breastfeeding duration (*n* = 163)		
		None (*n* = 44)	Partial (*n* = 66)	Exclusive (*n* = 48)	No (*n* = 17)	Yes (*n* = 146)	Never (*n* = 51)	<3 months (*n* = 54)	≥3 months (*n* = 58)
Acetate	79.9 (72.7–87.9)	77.3 (71.0–81.3)	79.4 (68.5–87.2)	86.7*** (77.0–95.5)	77.5 (73.2–81.7)	80.5 (72.3–89.4)	78.8 (71.0–86.0)	77.2 (70.1–83.9)	85.5* (75.6–94.9)
Butyrate	4.9 (1.5–7.7)	6.5 (4.5–8.2)	5.0 (2.1–7.6)	1.6*** (0.43–5.8)	6.4 (4.9–6.9)	4.0 (1.2–8.0)	5.6 (3.1–6.9)	6.2 (2.3–8.5)	1.9* (0.48–6.1)
Propionate	10.1 (4.4–16.8)	13.5 (7.1–19.0)	12.5 (5.4–19.5)	4.6*** (2.2–10.6)	13.4 (7.4–16.8)	9.9 (4.1–16.3)	12.4 (6.9–19.7)	13.9 (5.6–19.4)	5.7** (2.6–12.4)
Valerate	0.79 (0.41–1.46)	0.97 (0.63–1.6)	0.87 (0.36–1.4)	0.48** (0.24–1.3)	0.83 (0.60–1.5)	0.79 (0.36–1.4)	0.88 (0.41–1.6)	0.93 (0.60–1.4)	0.5 (0.25–1.3)
Isobutyrate	0.42 (0.18–0.91)	0.69 (0.39–1.2)	0.4 (0.23–1.0)	0.28*** (0.10–0.66)	0.85 (0.53–1.5)	0.37** (0.17–0.87)	0.64 (0.27–1.2)	0.48 (0.29–1.1)	0.27** (0.10–0.65)
Isovalerate	0.75 (0.25–1.5)	1.1 (0.82–1.8)	0.75* (0.29–1.6)	0.34*** (0.14–1.0)	1.56 (1.0–2.6)	0.64*** (0.22–1.4)	0.98 (0.42–1.8)	0.95 (0.59–1.8)	0.35** (0.17–1.0)

*Values are presented as median relative proportions (%) and IQR. Comparisons by non-parametric Mann–Whitney *U* test (two groups) or Kruskal–Wallis test (three groups, with Bonferroni posttest for multiple comparison) versus none or never breastfed category*.

***P < 0.001.

**P < 0.01.

*P < 0.05.

*^a^Breastfeeding status at fecal sample collection*.

**Figure 2:**
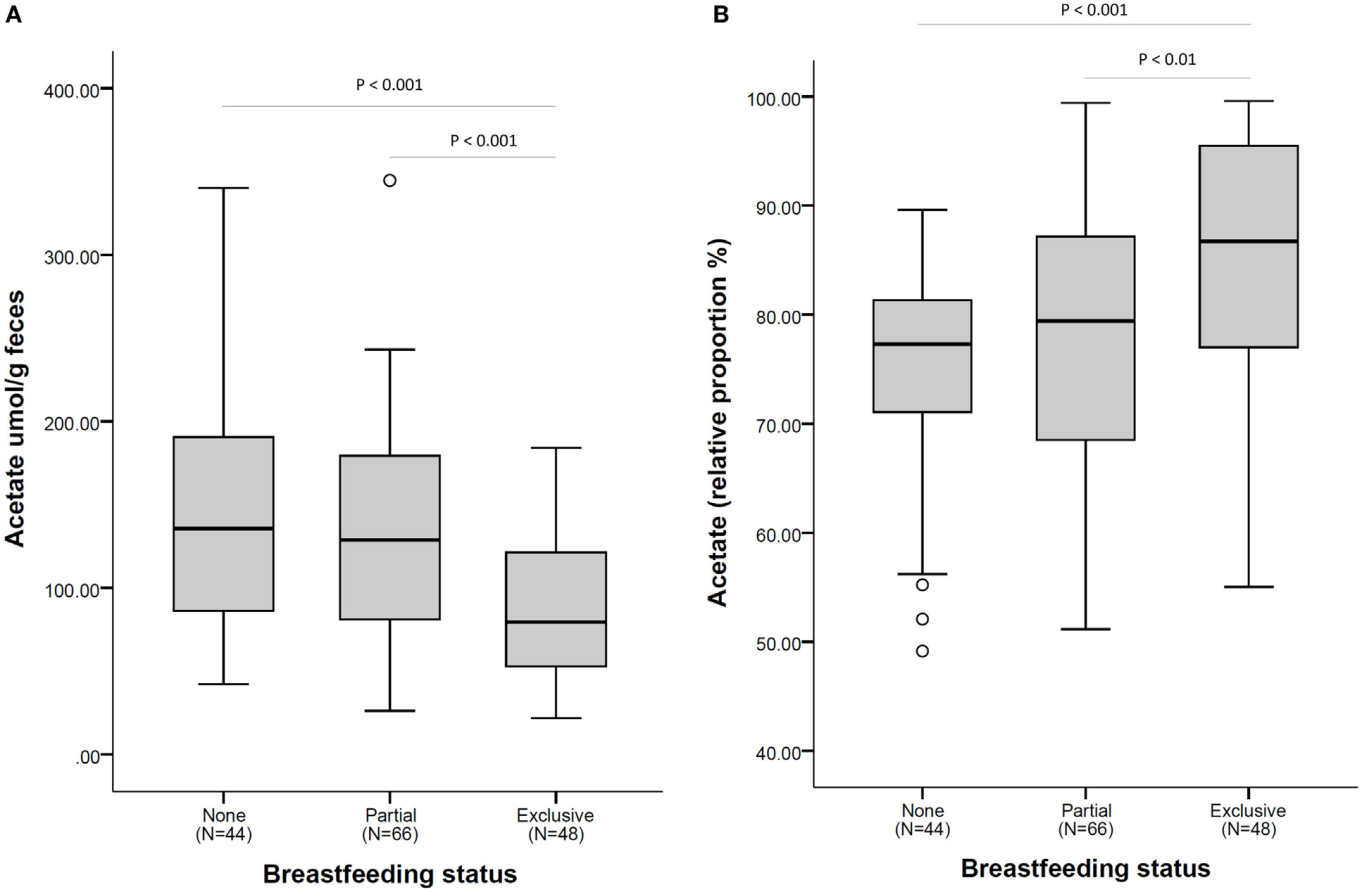
**Acetate fecal concentration (A) and as a relative proportion of total short-chain fatty acid (B), according to breastfeeding status at fecal sample collection**. *N* = 158. Comparisons by non-parametric Kruskal–Wallis test (with Bonferroni posttest for multiple comparison). Box plots present the group median (thick black line), upper quartile (top of box), and lower quartile (bottom of box). Whiskers present the maximum and minimum values excluding outliers (denoted by circles).

The higher relative proportion of acetate seen in exclusively breastfed infants is in contrast to the lower overall concentration of acetate in fecal samples of these infants as illustrated in Figure 2.

### Odds of High SCFAs and Intermediate Metabolites According to Breastfeeding, Adjusted for Birth Mode, and IAP

Exclusively breastfed infants had 86% lower odds of having high absolute concentrations of total SCFAs (concentrations greater than the group median) compared to those not breastfed at metabolite profiling [odds ratio (OR) 0.14, 95% CI 0.06–0.38; Table 5]. Odds of high total SCFAs was not significantly lower in those partially breastfed versus not breastfed (OR 0.92, 95% CI 0.42–2.05), although partial breastfeeding was associated with reduced odds of having high butyrate, valerate, isobutyrate, and isovalerate. Exclusively breastfed infants also had significantly reduced odds of having high acetate, butyrate, propionate, valerate, isobutyrate, and isovalerate compared to those not breastfed (Table 5). Similar results were seen in these infants following adjustment for birth mode and IAP, as well as age at stool collection, sex, city of birth, maternal education, and BMI (Table 5).

**Table 5:** **Associations between breastfeeding status and high metabolite concentrations, adjusting for birth mode and IAP, and other covariates**.

Breastfeeding status	Number of infants with high metabolite concentration, *n*/*N* (%) (*n* = 158)	*P* value for trend*	Odds of high metabolite concentration			
			Unadjusted OR (95% CI) (*n* = 153)	Model 1 aOR (95% CI) (*n* = 153)	Model 2 aOR (95% CI) (*n* = 153)	Model 3 aOR (95% CI) (*n* = 148)
**Total SCFA**						
None	28/44 (63.6)	<0.001	1.00 (ref)	1.00 (ref)	1.00 (ref)	1.00 (ref)
Partial	41/66 (62.1)		0.92 (0.42–2.05)	0.89 (0.40–2.01)	0.79 (0.34–1.83)	0.73 (0.30–1.75)
Exclusive	9/48 (18.8)		0.14 (0.06–0.38)	0.14 (0.05–0.36)	0.13 (0.05–0.38)	0.13 (0.04–0.39)
**Acetate**						
None	25/44 (56.8)	0.01	1.00 (ref)	1.00 (ref)	1.00 (ref)	1.00 (ref)
Partial	43/66 (65.2)		1.38 (0.62–3.05)	1.45 (0.64–3.30)	1.27 (0.54–3.00)	1.33 (0.54–3.28)
Exclusive	14/48 (29.2)		0.32 (0.13–0.75)	0.29 (0.12–0.71)	0.30 (0.11–0.79)	0.30 (0.11–0.86)
**Butyrate**						
None	33/44 (75.0)	<0.001	1.00 (ref)	1.00 (ref)	1.00 (ref)	1.00 (ref)
Partial	37/66 (56.1)		0.37 (0.15–0.87)	0.37 (0.16–0.90)	0.35 (0.14–0.87)	0.42 (0.16–1.11)
Exclusive	13/48 (27.1)		0.11 (0.04–0.28)	0.11 (0.04–0.29)	0.12 (0.04–0.35)	0.13 (0.04–0.39)
**Propionate**						
None	33/44 (75.0)	<0.001	1.00 (ref)	1.00 (ref)	1.00 (ref)	1.00 (ref)
Partial	40/66 (60.6)		0.50 (0.22–1.17)	0.45 (0.18–1.09)	0.42 (0.17–1.05)	0.48 (0.18–1.25)
Exclusive	8/48 (16.7)		0.07 (0.03–0.20)	0.68 (0.02–0.18)	0.06 (0.02–0.19)	0.07 (0.02–0.24)
**Valerate**						
None	32/44 (72.7)	<0.001	1.00 (ref)	1.00 (ref)	1.00 (ref)	1.00 (ref)
Partial	33/66 (50.0)		0.32 (0.14–0.75)	0.31 (0.13–0.74)	0.35 (0.14–0.86)	0.42 (0.16–1.07)
Exclusive	12/48 (25.0)		0.11 (0.04–0.28)	0.10 (0.04–0.27)	0.13 (0.04–0.36)	0.15 (0.05–0.45)
**Isobutyrate**						
None	34/44 (77.3)	<0.001	1.00 (ref)	1.00 (ref)	1.00 (ref)	1.00 (ref)
Partial	31/66 (47.0)		0.25 (0.11–0.59)	0.23 (0.09–0.55)	0.18 (0.07–0.47)	0.23 (0.09–0.63)
Exclusive	15/48 (31.3)		0.13 (0.05–0.34)	0.13 (0.05–0.35)	0.12 (0.04–0.33)	0.15 (0.05–0.45)
**Isovalerate**						
None	34/44 (77.3)	<0.001	1.00 (ref)	1.00 (ref)	1.00 (ref)	1.00 (ref)
Partial	34/66 (51.5)		0.30 (0.13–0.72)	0.30 (0.12–0.71)	0.25 (0.10–0.63)	0.33 (0.13–0.86)
Exclusive	11/25 (22.9)		0.08 (0.03–0.23)	0.09 (0.03–0.23)	0.08 (0.03–0.22)	0.09 (0.03–0.29)
**Lactate**						
None	10/44 (22.7)	<0.001	1.00 (ref)	1.00 (ref)	1.00 (ref)	1.00 (ref)
Partial	36/66 (54.5)		4.56 (1.88–11.04)	4.51 (1.82–11.18)	5.75 (2.16–15.26)	5.37 (1.90–15.15)
Exclusive	32/48 (66.7)		7.81 (2.99–20.37)	8.81 (3.27–23.72)	13.94 (4.39–44.24)	12.29 (3.64–41.46)
**Succinate**						
None	15/44 (34.1)	0.15	1.00 (ref)	1.00 (ref)	1.00 (ref)	1.00 (ref)
Partial	41/66 (62.1)		3.45 (1.53–7.79)	3.25 (1.42–7.41)	3.03 (1.30–7.02)	3.21 (1.31–7.88)
Exclusive	24/48 (50.0)		2.07 (0.88–4.90)	2.03 (0.85–4.84)	1.85 (0.72–4.72)	1.93 (0.71–5.22)

*Breastfeeding status at fecal sample collection. High metabolite concentration denotes values above the median. Model 1: adjusted for birth mode and IAP; model 2: adjusted for birth mode and IAP, age of stool collection, sex, and city of birth; model 3: adjusted for birth mode and IAP, age of stool collection, sex, city of birth, maternal BMI, and education*.

*OR, odds ratio; SCFA, short-chain fatty acid; IAP, intrapartum antibiotic prophylaxis*.

*Pearson chi-square *P* value for linear trend (univariate).

Exclusively breastfed infants were seven times more likely to have high concentrations of lactate, and partially breastfed infants four times more likely than those not breastfed (OR 7.81, 95% CI 2.99–20.37 and OR 4.56, 95% CI 1.88–11.04, respectively; Table 5). These associations remained after adjustment for covariates. Partially breastfed infants, but not exclusively breastfed infants, had significantly higher odds of high succinate (OR 3.45, 95% CI 1.53–7.79).

Infants exclusively breastfed were over four times more likely to have high relative proportions of acetate compared to those not breastfed (OR 4.26, 95% CI 1.76–10.36; Table 6). This association was independent of birth mode and IAP, as well as other covariates (aOR 4.50, 95% CI 1.58–12.82). In line with this, odds of high butyrate, propionate, valerate, isobutyrate, and isovalerate were significantly lower in exclusively breastfed infants. Associations with valerate did not remain after adjustment with all covariates. Partial breastfeeding was associated with reduced odds of high butyrate (OR 0.34, 95% CI 0.15–0.80), and isovalerate (OR 0.36, 95% CI 0.16–0.81) although these associations were no longer significant after additional adjustment for maternal education and BMI.

**Table 6:** **Associations between breastfeeding status and high relative proportions of SCFA, adjusting for birth mode and IAP, and other covariates**.

Breastfeeding status	Number of infants with high relative proportion of metabolite, *n*/*N* (%) (*n* = 158)	*P* value for trend*	Odds of high relative proportion of metabolite			
			Unadjusted OR (95% CI) (*n* = 153)	Model 1 aOR (95% CI) (*n* = 153)	Model 2 aOR (95% CI) (*n* = 153)	Model 3 aOR (95% CI) (*n* = 148)
**Acetate**						
None	15/44 (34.1)	<0.001	1.00 (ref)	1.00 (ref)	1.00 (ref)	1.00 (ref)
Partial	35/66 (53.0)		2.25 (1.02–5.00)	2.25 (0.99–5.09)	2.18 (0.94–5.09)	2.12 (0.86–5.23)
Exclusive	34/48 (70.8)		4.26 (1.76–10.36)	4.38 (1.78–10.83)	5.28 (1.96–14.26)	4.50 (1.58–12.82)
**Butyrate**						
None	32/44 (72.7)	<0.001	1.00 (ref)	1.00 (ref)	1.00 (ref)	1.00 (ref)
Partial	34/66 (51.5)		0.34 (0.15–0.80)	0.33 (0.14–0.78)	0.35 (0.14–0.87)	0.51 (0.19–1.35)
Exclusive	16/48 (33.3)		0.17 (0.07–0.42)	0.17 (0.07–0.43)	0.17 (0.06–0.47)	0.22 (0.07–0.65)
**Propionate**						
None	27/44 (61.4)	0.001	1.00 (ref)	1.00 (ref)	1.00 (ref)	1.00 (ref)
Partial	37/66 (56.1)		0.79 (0.36–1.73)	0.79 (0.35–1.79)	0.68 (0.29–1.59)	0.72 (0.29–1.77)
Exclusive	13/48 (27.1)		0.26 (0.11–0.63)	0.24 (0.10–0.60)	0.21 (0.08–0.56)	0.25 (0.09–0.71)
**Valerate**						
None	25/44 (56.8)	0.04	1.00 (ref)	1.00 (ref)	1.00 (ref)	1.00 (ref)
Partial	32/66 (48.5)		0.64 (0.29–1.39)	0.58 (0.26–1.30)	0.65 (0.28–1.50)	0.84 (0.35–2.06)
Exclusive	17/48 (35.4)		0.38 (0.16–0.91)	0.39 (0.16–0.92)	0.44 (0.17–1.12)	0.58 (0.21–1.58)
**Isobutyrate**						
None	30/44 (68.2)	0.002	1.00 (ref)	1.00 (ref)	1.00 (ref)	1.00 (ref)
Partial	33/66 (50.0)		0.45 (0.20–1.01)	0.42 (0.19–0.97)	0.37 (0.16–0.88)	0.53 (0.21–1.33)
Exclusive	17/48 (35.4)		0.26 (0.11–0.62)	0.26 (0.11–0.64)	0.22 (0.08–0.58)	0.30 (0.11–0.85)
**Isovalerate**						
None	32/44 (72.7)	<0.001	1.00 (ref)	1.00 (ref)	1.00 (ref)	1.00 (ref)
Partial	31/66 (47.0)		0.32 (0.14–0.73)	0.33 (0.14–0.77)	0.33 (0.14–0.78)	0.45 (0.18–1.09)
Exclusive	13/48 (27.1)		0.14 (0.05–0.35)	0.13 (0.05–0.34)	0.13 (0.05–0.35)	0.16 (0.06–0.47)

*Breastfeeding status at fecal sample collection. High metabolite proportion denotes values above the median. Model 1: adjusted for birth mode and IAP; model 2: adjusted for birth mode and IAP, age of stool collection, sex, and city of birth; model 3: adjusted for birth mode and IAP, age of stool collection, sex, city of birth, maternal BMI, and education*.

*OR, odds ratio; SCFA, short-chain fatty acid; IAP, intrapartum antibiotic prophylaxis*.

*Pearson chi-square *P* value for linear trend (univariate).

## Discussion

In this subsample of 163 Canadian infants aged between 3 and 5 months from a general population birth cohort, we found strong associations between breastfeeding exclusivity and infant fecal metabolites. Exclusive breastfeeding was associated with lower absolute concentrations of total SCFAs, acetate, butyrate, propionate, valerate, isobutyrate, and isovalerate, yet higher concentrations of lactate. Further, the relative proportion of acetate was higher with exclusive breastfeeding. Compared to non-breastfed infants, those exclusively breastfed were four times more likely (aOR 4.50, 95% CI 1.58–12.82) to have a higher proportion of acetate relative to other SCFAs in their gut. This association was independent of birth mode, intrapartum antibiotics, infant sex, age, recruitment site, and maternal BMI or socioeconomic status.

Our results are similar to that of Edwards et al. who also found a predominance in relative proportions of acetate (76%) in breastfed infants at 4 weeks of age (19). Higher relative abundance of acetate in exclusively breastfed infants may in part be due to the presence of HMOs in breast milk, not present in formula. HMOs, which represent the third largest component of breast milk, are soluble carbohydrates that are undigested by the host and provide substrates for select gut microbiota (27). *Bifidobacterium* species have been shown to be one of a narrow selection of gut bacteria that are able to metabolize HMO (28) and subsequently are overrepresented in the microbiota of breastfed infants compared to formula-fed infants (11, 16, 29). Results from our group by Azad et al. found the *Bifidobacteriaceae* family to be enriched with breastfeeding (11). Bifidobacterium have been shown to metabolize HMO to produce acetate and lactate (30, 31). Martin et al. observed that increases in bifidobacteria counts corresponded to increases in fecal acetate (12). Although evidence is limited, higher acetate in breastfed infants may afford protection against intestinal pathogens and allergic disease (3). Fukudo et al. demonstrated that acetate, produced by bifidobacteria, improved intestinal defense and protected against *Escherichia coli* O157:H7 in mice (30). Thornburn et al. have proposed that acetate-mediated inhibition of histone deacetylases, demonstrated in an adult mouse model, increases transcription of the Foxp3 gene that may promote Treg-suppressing airway inflammation and inducing oral tolerance. In infants, Arrieta et al. found that reduced fecal acetate at 3 months was associated with allergic disease in later infancy (32).

We also observed lower absolute concentrations of SCFAs in exclusively breastfed infants, which are consistent with other published studies. In 111 fecal samples analyzed by NMR, Martin et al. found lower concentrations of SCFAs in breastfed infants at 3 and 6 months born to overweight or obese mothers (20). In a small study on 4 infants using GC and LC mass spectrometry, valerate and isovalerate concentrations were higher in formula-fed infants, the latter over 40 times higher than breastfed infants (18). A study of 67 infants not only found lower fecal SCFA concentrations in breastfed infants but also observed that the addition of milk (formula or cows milk) to the diet of breastfed infants was sufficient to change the SCFA profile (22). We observed similar results, in that partially breastfed infants had SCFA and lactate concentrations more similar to exclusively formula-fed infants than infants exclusively breastfed.

The higher absolute concentrations of SCFAs observed in formula-fed infants may be a consequence of greater bacterial diversity observed in these infants in comparison to breastfed fed infants (11) and thus increased capability to metabolize substrates present in the gut. Indeed, previous results from our group found breastfeeding exclusivity to be inversely associated with both microbiota richness and diversity (11). Our finding may also be a result of differences in the composition or absorption of breast milk versus formula milk and thus differences in availability of substrates. Further, higher concentrations of branch-chain fatty acids, valerate, isobutyrate and isovalerate, derived from the metabolism of amino acids indicate reduced protein absorption or excess protein intake [potentially due to higher protein content of formula versus human milk (33)] in formula-fed infants. The availability of these substrates likely drive the higher abundance of proteolytic bacteria such as Bacteroides and Clostridia seen in formula-fed infants as reported in our study by Azad et al. (11). Higher concentrations of proteolytic metabolites in formula-fed infants may also be due to reduced carbohydrate availability in the absence of HMOs and hence to greater derivation of energy from protein metabolism. Chow et al. demonstrated that when fermentable carbohydrates were not present in fecal cultures from both breast and formula-fed infants, metabolites indicative of protein fermentation were mainly produced; their production was reduced after the addition of various fermentable carbohydrate substrates similar to HMOs. Increased fecal SCFAs seen in formula-fed infants may have metabolic consequences. Several studies have reported greater concentrations of fecal SCFAs in overweight adults and children compared to lean counterparts (34–38) and correlations with other metabolic risk factors (38). Although causality is yet to be established, the authors of these studies hypothesize that higher SCFAs may reflect an increased capacity of the gut microbiota to harvest energy from the diet.

In addition to SCFAs, we also measured concentrations of lactate and succinate. Both are important intermediates in the production of SCFAs (3). Succinate is primarily converted to propionate through the succinate pathway. Lactate, produced by many colonizers of the infant gut including bifidobacteria and lactobacilli, can also be converted to propionate through the acrylate pathway but is also an important substrate used in cross-feeding by lactate-utilizing bacteria in the production of SCFAs as demonstrated by Pham et al. (24). Similar to other studies (18–20, 22), we observed higher lactate concentrations in exclusively breastfed infants, which is indicative of the predominance of lactate-producing bacteria, *Lactobacillus* and bifidobacteria, in the gut of breastfed infants. Lactate production in the gut affects luminal pH, and stool from breastfed infants typically has a lower fecal pH compared to formula-fed infants (5.8 versus 6.3–7.10) (22, 39). Studies in adults have suggested that accumulation of lactate in the gut may have undesirable health consequences including increased risk of ulcerative colitis; however, in infancy, it may be an important mechanism in preventing overgrowth of pH-sensitive pathogenic bacteria, such as Enterobacteriaceae and Clostridia (40, 41). Lactate has also been shown to be important in modulating immune and inflammatory processes and maintaining gut barrier function through stimulation of enterocyte proliferation (42). We are the first to report on differences in fecal succinate in infants. Although understudied, succinate may be an important signaling molecule and has been shown to activate GPR91 on dendritic cells and thus may play a role in modulation of gut immunity and inflammation (6, 43). Unlike the other associations we observed, differences in succinate were only apparent in those partially breastfed. This observation may in part be explained by the higher abundance of Bacteroidetes observed in partially breastfed infants reported in our study infants by Azad et al (11). Bacteroidetes utilize the succinate pathway in the formation of propionate (4, 44).

One of the strengths of our study is the use of infants from a well-characterized general birth cohort, affording detailed information on birth and early-life characteristics. This, along with our relatively large sample size, enabled us to account for prenatal and postnatal factors shown to impact early gut microbiota development (most importantly birth mode and perinatal antibiotic exposure) in our estimates of associations between fecal metabolites and breastfeeding. While we adjusted for IAP, shown to be significantly associated with gut microbiota dysbiosis in early life (11), we did not additionally adjust for postnatal infant antibiotic use due to missing data in our sample. However, it has been shown that IAP is a good proxy for perinatal antibiotic use in infancy (11). One limitation of our study was the indirect measurement of luminal metabolites *via* analysis of fecal samples. Concentrations of metabolites in feces are a function not only of production but also of absorption, utilization by other microbes and stool transit time. Few studies have estimated whether fecal SCFA output is a reliable proxy for luminal content, particularly in early childhood although it is estimated that 95% of the SCFAs produced in the gut are rapidly absorbed with only 5% being excreted in the feces (45). In a study on healthy adults, Vogt and Wolever found fecal acetate to be inversely correlated to acetate absorption, suggesting that fecal acetate concentration may reflect intestinal absorption rather than production (46). However, given that the potential of analyzing fecal metabolites is to provide a biomarker to predict future disease risk, analysis of fecal samples provides a non-invasive and cost-effective method for use in epidemiological cohort studies. Our sampling was also only at one time point in infancy, and therefore, our data do not provide information on trends in metabolites over time in relation to diet.

**Figure 3:**
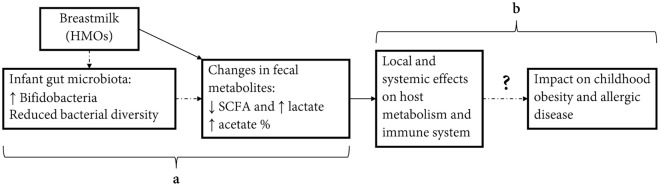
**Effect of infant diet on fecal short-chain fatty acids (SCFAs) and potential effect on host metabolic and immune programming**. Human milk feeding causes changes in infant fecal metabolites, potentially through effects on gut microbiota composition (a). Changes in concentrations and relative proportions of SCFA and intermediate metabolites exert local effects on the gut environment and act as signaling molecules effecting host metabolism and immune system. These actions may have important programming effects on inflammatory-mediated diseases in childhood, including obesity and allergic disease (b).

In conclusion, exclusive breastfeeding has been associated with a number of beneficial health outcomes in early childhood including reduced infections, allergic disease, and improved metabolic markers (1). Recent research has highlighted the importance of our gut microbiota and associated metabolites as a potential causal factor or mediator in the programming of later disease states. Our study confirms that breastfeeding is strongly associated with gut metabolites, which may be an important mediator in the protective effect of breastfeeding on later-onset diseases (Figure 3). Given differences seen in the study between metabolites measured as absolute concentrations and relative proportions, we encourage future studies to report on both of these measures in relation to health outcomes.

## Ethics Statement

Written informed consent was obtained from parents at enrollment. This study was approved by the University of Alberta, University of British Columbia and University of Manitoba, and McMaster University Human Research Ethics Boards.

## Author Contributions

AK, SB, MA, CF, AH, PM, and DW conceptualized the research question. SB analyzed the data and drafted the manuscript. SB and AK interpreted findings and edited the manuscript. MA created breastfeeding measures for use in the analysis, contributed to interpretation of findings, and edited the manuscript. DW conducted NMR analysis on the fecal samples. JS, MS, AB, PM, PS, and ST directed the design and implementation of the CHILD study from which these samples were drawn. All authors revised and approved the final manuscript.

## Conflict of Interest Statement

The authors declare that the research was conducted in the absence of any commercial or financial relationships that could be construed as a potential conflict of interest.

## References

[1] Victora CG Bahl R Barros AJ Franca GV Horton S Krasevec J Breastfeeding in the 21st century: epidemiology, mechanisms, and lifelong effect. Lancet (2016) 387:475–90. 10.1016/S0140-6736(15)01024-7 26869575

[2] Sommer F Backhed F The gut microbiota – masters of host development and physiology. Nat Rev Microbiol (2013) 11:227–38. 10.1038/nrmicro2974 23435359

[3] Kumari M Kozyrskyj AL Gut microbial metabolism defines host metabolism: an emerging perspective in obesity and allergic inflammation. Obes Rev (2017) 18:18–31. 10.1111/obr.12484 27862824

[4] Louis P Flint HJ Formation of propionate and butyrate by the human colonic microbiota. Environ Microbiol (2016) 1:1 10.1111/1462-2920.13589 27928878

[5] Bergman EN. Energy contributions of volatile fatty acids from the gastrointestinal tract in various species. Physiol Rev (1990) 70:567–90. 2181501 10.1152/physrev.1990.70.2.567

[6] Koh A De Vadder F Kovatcheva-Datchary P Backhed F From dietary fiber to host physiology: short-chain fatty acids as key bacterial metabolites. Cell (2016) 165:1332–45. 10.1016/j.cell.2016.05.041 27259147

[7] Morrison DJ Preston T. Formation of short chain fatty acids by the gut microbiota and their impact on human metabolism. Gut Microbes (2016) 7:189–200. 10.1080/19490976.2015.1134082 26963409 PMC4939913

[8] Correa-Oliveira R Fachi JL Vieira A Sato FT Vinolo MA. Regulation of immune cell function by short-chain fatty acids. Clin Transl Immunol (2016) 5:e73. 10.1038/cti.2016.17 27195116 PMC4855267

[9] Richards JL Yap YA McLeod KH Mackay CR Marino E. Dietary metabolites and the gut microbiota: an alternative approach to control inflammatory and autoimmune diseases. Clin Transl Immunol (2016) 5:e82. 10.1038/cti.2016.29 27350881 PMC4910123

[10] Flint HJ Duncan SH Scott KP Louis P. Links between diet, gut microbiota composition and gut metabolism. Proc Nutr Soc (2015) 74:13–22. 10.1017/S0029665114001463 25268552

[11] Azad MB Konya T Persaud RR Guttman DS Chari RS Field CJ Impact of maternal intrapartum antibiotics, method of birth and breastfeeding on gut microbiota during the first year of life: a prospective cohort study. BJOG (2016) 123:983–93. 10.1111/1471-0528.13601 26412384

[12] Martin R Makino H Yavuz AC Ben-Amor K Roelofs M Ishikawa E Early-life events, including mode of delivery and type of feeding, siblings and gender, shape the developing gut microbiota. PLoS One (2016) 11:e0158498. 10.1371/journal.pone.0158498 27362264 PMC4928817

[13] Yassour M Vatanen T Siljander H Hamalainen AM Harkonen T Ryhanen SJ Natural history of the infant gut microbiome and impact of antibiotic treatment on bacterial strain diversity and stability. Sci Transl Med (2016) 8:343ra81. 10.1126/scitranslmed.aad0917 27306663 PMC5032909

[14] Bode L The functional biology of human milk oligosaccharides. Early Hum Dev (2015) 91:619–22. 10.1016/j.earlhumdev.2015.09.001 26375354

[15] Azad MB Konya T Maughan H Guttman DS Field CJ Chari RS Gut microbiota of healthy Canadian infants: profiles by mode of delivery and infant diet at 4 months. CMAJ (2013) 185:385–94. 10.1503/cmaj.121189 23401405 PMC3602254

[16] Backhed F Roswall J Peng Y Feng Q Jia H Kovatcheva-Datchary P Dynamics and stabilization of the human gut microbiome during the first year of life. Cell Host Microbe (2015) 17:690–703. 10.1016/j.chom.2015.04.004 25974306

[17] Penders J Thijs C Vink C Stelma FF Snijders B Kummeling I Factors influencing the composition of the intestinal microbiota in early infancy. Pediatrics (2006) 118:511–21. 10.1542/peds.2005-2824 16882802

[18] Chow J Panasevich MR Alexander D Vester Boler BM Rossoni Serao MC Faber TA Fecal metabolomics of healthy breast-fed versus formula-fed infants before and during in vitro batch culture fermentation. J Proteome Res (2014) 13:2534–42. 10.1021/pr500011w 24628373

[19] Edwards CA Parrett AM Balmer SE Wharton BA. Faecal short chain fatty acids in breast-fed and formula-fed babies. Acta Paediatr (1994) 83:459–62. 10.1111/j.1651-2227.1994.tb13059.x 8086719

[20] Martin FP Moco S Montoliu I Collino S Da Silva L Rezzi S Impact of breast-feeding and high- and low-protein formula on the metabolism and growth of infants from overweight and obese mothers. Pediatr Res (2014) 75:535–43. 10.1038/pr.2013.250 24375085

[21] Midtvedt AC Midtvedt T. Production of short chain fatty acids by the intestinal microflora during the first 2 years of human life. J Pediatr Gastroenterol Nutr (1992) 15:395–403. 10.1097/00005176-199211000-00005 1469519

[22] Ogawa K Ben RA Pons S de Paolo MI Bustos Fernandez L. Volatile fatty acids, lactic acid, and pH in the stools of breast-fed and bottle-fed infants. J Pediatr Gastroenterol Nutr (1992) 15:248–52. 10.1097/00005176-199210000-00004 1432461

[23] Siigur U Ormisson A Tamm A. Faecal short-chain fatty acids in breast-fed and bottle-fed infants. Acta Paediatr (1993) 82:536–8. 10.1111/j.1651-2227.1993.tb12747.x 8338985

[24] Pham VT Lacroix C Braegger CP Chassard C Early colonization of functional groups of microbes in the infant gut. Environ Microbiol (2016) 18:2246–58. 10.1111/1462-2920.13316 27059115

[25] Subbarao P Anand SS Becker AB Befus AD Brauer M Brook JR The Canadian Healthy Infant Longitudinal Development (CHILD) study: examining developmental origins of allergy and asthma. Thorax (2015) 70:998–1000. 10.1136/thoraxjnl-2015-207246 26069286

[26] Matysik S Le Roy CI Liebisch G Claus SP Metabolomics of fecal samples: a practical consideration. Trends Food Sci Technol (2016) 57:244–55. 10.1016/j.tifs.2016.05.011

[27] Garrido D Dallas DC Mills DA. Consumption of human milk glycoconjugates by infant-associated bifidobacteria: mechanisms and implications. Microbiology (2013) 159:649–64. 10.1099/mic.0.064113-0 23460033 PMC4083661

[28] Garrido D Ruiz-Moyano S Lemay DG Sela DA German JB Mills DA Comparative transcriptomics reveals key differences in the response to milk oligosaccharides of infant gut-associated bifidobacteria. Sci Rep (2015) 5:13517 10.1038/srep13517 26337101 PMC4559671

[29] Harmsen HJ Wildeboer-Veloo AC Raangs GC Wagendorp AA Klijn N Bindels JG Analysis of intestinal flora development in breast-fed and formula-fed infants by using molecular identification and detection methods. J Pediatr Gastroenterol Nutr (2000) 30:61–7. 10.1097/00005176-200001000-00019 10630441

[30] Fukuda S Toh H Hase K Oshima K Nakanishi Y Yoshimura K Bifidobacteria can protect from enteropathogenic infection through production of acetate. Nature (2011) 469:543–7. 10.1038/nature09646 21270894

[31] Matsuki T Yahagi K Mori H Matsumoto H Hara T Tajima S A key genetic factor for fucosyllactose utilization affects infant gut microbiota development. Nat Commun (2016) 7:11939. 10.1038/ncomms11939 27340092 PMC4931012

[32] Arrieta MC Stiemsma LT Dimitriu PA Thorson L Russell S Yurist-Doutsch S Early infancy microbial and metabolic alterations affect risk of childhood asthma. Sci Transl Med (2015) 7:307ra152. 10.1126/scitranslmed.aab2271 26424567

[33] Mace K Steenhout P Klassen P Donnet A. Protein quality and quantity in cow’s milk-based formula for healthy term infants: past, present and future. Nestle Nutr Workshop Ser Pediatr Program (2006) 58:109–203. 10.1159/000095063 16902335

[34] Payne AN Chassard C Zimmermann M Muller P Stinca S Lacroix C. The metabolic activity of gut microbiota in obese children is increased compared with normal-weight children and exhibits more exhaustive substrate utilization. Nutr Diabetes (2011) 1:e12. 10.1038/nutd.2011.8 23154580 PMC3302137

[35] Fernandes J Su W Rahat-Rozenbloom S Wolever TM Comelli EM. Adiposity, gut microbiota and faecal short chain fatty acids are linked in adult humans. Nutr Diabetes (2014) 4:e121. 10.1038/nutd.2014.23 24979150 PMC4079931

[36] Rahat-Rozenbloom S Fernandes J Gloor GB Wolever TM. Evidence for greater production of colonic short-chain fatty acids in overweight than lean humans. Int J Obes (Lond) (2014) 38:1525–31. 10.1038/ijo.2014.46 24642959 PMC3970979

[37] Schwiertz A Taras D Schafer K Beijer S Bos NA Donus C Microbiota and SCFA in lean and overweight healthy subjects. Obesity (Silver Spring) (2010) 18:190–5. 10.1038/oby.2009.167 19498350

[38] Teixeira TF Grzeskowiak L Franceschini SC Bressan J Ferreira CL Peluzio MC. Higher level of faecal SCFA in women correlates with metabolic syndrome risk factors. Br J Nutr (2013) 109:914–9. 10.1017/S0007114512002723 23200109

[39] Knol J Scholtens P Kafka C Steenbakkers J Gro S Helm K Colon microflora in infants fed formula with galacto- and fructo-oligosaccharides: more like breast-fed infants. J Pediatr Gastroenterol Nutr (2005) 40:36–42. 10.1097/00005176-200501000-00007 15625424

[40] Duncan SH Louis P Thomson JM Flint HJ. The role of pH in determining the species composition of the human colonic microbiota. Environ Microbiol (2009) 11:2112–22. 10.1111/j.1462-2920.2009.01931.x 19397676

[41] Sun Y O’Riordan MX. Regulation of bacterial pathogenesis by intestinal short-chain fatty acids. Adv Appl Microbiol (2013) 85:93–118. 10.1016/B978-0-12-407672-3.00003-4 23942149 PMC4029053

[42] Garrote GL Abraham AG Rumbo M. Is lactate an undervalued functional component of fermented food products? Front Microbiol (2015) 6:629. 10.3389/fmicb.2015.00629 26150815 PMC4473639

[43] Rubic T Lametschwandtner G Jost S Hinteregger S Kund J Carballido-Perrig N Triggering the succinate receptor GPR91 on dendritic cells enhances immunity. Nat Immunol (2008) 9:1261–9. 10.1038/ni.1657 18820681

[44] Louis P Hold GL Flint HJ The gut microbiota, bacterial metabolites and colorectal cancer. Nat Rev Microbiol (2014) 12:661–72. 10.1038/nrmicro3344 25198138

[45] den Besten G van Eunen K Groen AK Venema K Reijngoud DJ Bakker BM. The role of short-chain fatty acids in the interplay between diet, gut microbiota, and host energy metabolism. J Lipid Res (2013) 54:2325–40. 10.1194/jlr.R036012 23821742 PMC3735932

[46] Vogt JA Wolever TM. Fecal acetate is inversely related to acetate absorption from the human rectum and distal colon. J Nutr (2003) 133:3145–8. 14519799 10.1093/jn/133.10.3145

