# Supplementary material for: Fecal Short-Chain Fatty Acid Variations by Breastfeeding Status in Infants at 4 Months: Differences in Relative versus Absolute Concentrations
Source: Front Nutr. 2017 Apr 10;4:11. doi: 10.3389/fnut.2017.00011 (PMC5385454; doi:10.3389/fnut.2017.00011)
Supplement: Supplementary file 1 [file Table_1.DOCX]

**Supplementary Data**

Table S1. Median concentrations of SCFA and intermediate metabolites according to breastfeeding status and duration restricted to infants not introduced to solid foods.

|  |  | **Breastfeeding status† n=133** | | | **Ever breastfed n=137** | | **Exclusive breastfeeding duration n=137** | | |
| --- | --- | --- | --- | --- | --- | --- | --- | --- | --- |
| **Metabolite μmol/g (IQR)** | **All infants (n=137)** | **None (n=31)** | **Partial (n=54)** | **Exclusive (n=48)** | **No (n=14)** | **Yes (n=123)** | **Never (n=43)** | **<3 months (n=43)** | **≥3 months (n=51)** |
| Total SCFA‡ | 145.1 (97.9-200.6) | 194.1 (133.0-244.8) | 167.2 (130.4-234.7) | 99.4*** (63.8-130.5) | 181.7 (135.1-236.3) | 139.0 (96.0-200.0) | 178.4 (143.6-239.9) | 171.2 (119.6-229.1) | 100.8*** (63.8-132.7) |
| Acetate | 119.0 (74.5-165.2) | 144.3 (105.1-190.5) | 137.8 (81.3-185.6) | 79.4*** (52.7-121.3) | 126.8 (109.4-193.9) | 116.5 (73.6-164.9) | 146.5 (101.5-200.8) | 124.4 (83.6-172.5) | 80.2*** (52.7-125.3) |
| Butyrate | 6.3 (1.9-12.5) | 13.2 (8.3-19.0) | 7.0** (3.3-10.6) | 1.6*** (0.44-7.2) | 10.9 (6.1-15.8) | 5.2* (1.6-12.0) | 9.2 (5.1-13.7) | 7.0 (3.7-16.3) | 1.7*** (0.44-7.3) |
| Propionate | 13.1 (4.3-27.9) | 19.8 (10.5-35.8) | 21.6 (9.6-35.8) | 4.3*** (1.7-11.1) | 20.8 (15.1-30.0) | 11.6 (4.2-27.3) | 22.5 (13.5-34.1) | 15.2 (9.2-35.6) | 4.3*** (1.7-11.5) |
| Valerate | 1.2 (0.41-2.4) | 2.3 (1.5-3.2) | 1.4 (0.64-2.5) | 0.39*** (0.15-1.2) | 1.8 (1.1-3.3) | 1.2 (0.37-2.3) | 1.8 (0.77-3.0) | 1.7 (0.88-2.8) | 0.41*** (0.17-1.3) |
| Isobutyrate | 0.67 (0.21-1.7) | 1.4 (0.76-2.2) | 0.64 (0.31-2.0) | 0.20*** (0.07-0.72) | 1.5 (0.77-3.3) | 0.57** (0.20-1.7) | 1.3 (0.62-1.9) | 0.64 (0.40-2.1) | 0.21*** (0.07-0.72) |
| Isovalerate | 1.0 (0.31-2.4) | 2.2 (1.6-3.8) | 0.13* (0.48-3.1) | 0.33*** (0.09-0.91) | 3.4 (1.8-7.0) | 0.92*** (0.28-2.0) | 1.8 (0.79-3.3) | 1.5 (0.68-3.4) | 0.34*** (0.09-1.1) |
| Lactate | 3.6 (1.5-19.3) | 1.9 (1.2-2.8) | 4.0* (1.5-12.6) | 7.2*** (2.2-32.7) | 2.2 (1.3-3.5) | 4.3 (1.5-21.5) | 3.1 (1.5-11.5) | 2.5 (1.2-7.2) | 6.7 (2.3-31.2) |
| Succinate | 8.3 (3.2-28.7) | 4.2 (1.8-18.4) | 15.7* (3.8-57.7) | 8.4 (3.4-30.6) | 3.2 (1.4-9.5) | 10.4* (3.5-33.7) | 9.5 (3.0-25.9) | 8.3 (3.3-31.3) | 7.1 (3.2-30.6) |

Values are presented as medians and interquartile ranges (IQR) in μmol/g feces. Comparisons by non-parametric Mann Whitney U test (2 groups ) or Kruskal Wallis test (3 groups, with Bonferroni post-test for multiple comparison) versus none or never breastfeeding category. *** p<0.001, ** p<0.01, * p<0.05. †Breastfeeding status as stool sample collection.‡Total SCFA represent the sum of acetate, butyrate, propionate, valerate, isobutyrate, and isovalerate.

Table S2. Relative proportions of total SCFA according to breastfeeding status and duration restricted to infants not introduced to solid foods.

|  |  | **Breastfeeding status† n=133** | | | **Ever breastfed n=137** | | **Exclusive breastfeeding duration n=137** | | |
| --- | --- | --- | --- | --- | --- | --- | --- | --- | --- |
| **Metabolite % (IQR)** | **All infants (n=137)** | **None (n=31)** | **Partial (n=54)** | **Exclusive (n-48)** | **No (n=14)** | **Yes (n=123)** | **Never (n=43)** | **<3 months (n=43)** | **≥3 months (n=51)** |
| Acetate | 80.6 (73.2-89.4) | 77.1 (73.4-81.6) | 79.4 (64.9-87.8) | 86.7** (77.0-95.5) | 79.0 (73.2-82.0) | 80.6 (73.0-91.3) | 78.8 (71.5-85.8) | 76.8 (67.8-85.0) | 86.1** (77.0-95.3) |
| Butyrate | 4.2 (1.3-7.6) | 6.7 (5.0-8.7) | 3.9* (2.0-7.6) | 1.6*** (0.43-5.8) | 6.5 (4.9-6.9) | 3.8 (1.2-7.8) | 5.3 (3.1-6.9) | 6.3 (2.3-8.9) | 1.7* (0.46-5.8) |
| Propionate | 9.9 (3.9-15.8) | 11.8 (6.9-15.2) | 14.5 (5.3-21.6) | 4.6* (2.2-10.6) | 11.3 (7.1-18.8) | 9.4 (3.8-15.8) | 11.6 (6.9-19.7) | 13.8 (5.0-19.9) | 4.6** (2.5-11.3) |
| Valerate | 0.84 (0.41-1.5) | 1.3 (0.78-1.8) | 0.87 (0.39-1.4) | 0.48** (0.24-1.3) | 0.89 (0.60-1.8) | 0.79 (0.37-1.5) | 1.0 (0.44-1.7) | 0.99 (0.58-1.5) | 0.50 (0.26-1.2) |
| Isobutyrate | 0.42 (0.18-0.89) | 0.79 (0.42-1.4) | 0.44 (0.23-1.0) | 0.28** (0.10-0.66) | 0.84 (0.53-1.5) | 0.37* (0.16-0.87) | 0.64 (0.29-1.2) | 0.45 (0.27-1.0) | 0.28** (0.10-0.64) |
| Isovalerate | 0.74 (0.22-1.6) | 1.1 (0.87-2.0) | 0.77 (0.29-1.8) | 0.34*** (0.14-1.0) | 1.8 (1.1-2.8) | 0.62*** (0.21-1.5) | 0.99 (0.46-1.8) | 0.95 (0.59-1.8) | 0.38** (0.15-1.0) |

Values are presented as median relative proportions (%) and interquartile range (IQR). Comparisons by non-parametric Mann Whitney U test (2 groups) or Kruskal Wallis test (3 groups, with Bonferroni post-test for multiple comparison) versus none or never breastfed category. *** p<0.001, ** p<0.01, * p<0.05. †Breastfeeding status at fecal sample collection.

Table S3. Median concentrations of SCFA and intermediate metabolites according to breastfeeding status and duration restricted to vaginally delivered infants not receiving IAP.

|  |  | **Breastfeeding status† n=76** | | | **Ever breastfed n=78** | | **Exclusive breastfeeding duration n=78** | | |
| --- | --- | --- | --- | --- | --- | --- | --- | --- | --- |
| **Metabolite μmol/g (IQR)** | **All infants (n=78)** | **None (n=24)** | **Partial (n=26)** | **Exclusive (n=26)** | **No (n=11)** | **Yes (n=67)** | **Never (n=21)** | **<3 months (n=25)** | **≥3 months (n=32)** |
| Total SCFA‡ | 134.5 (97.9-197.4) | 181.7 (120.3-249.2) | 150.7 (108.4-239.4) | 108.8** (70.6-148.4) | 185.0 (123.4-252.5) | 131.9 (96.9-192.2) | 178.4 (133.0-265.2) | 153.2 (108.4-212.6) | 101.9*** (65.9-153.4) |
| Acetate | 112.1 (73.2-159.3) | 119.4 (86.2-188.1) | 124.8 (76.3-171.4) | 92.9* (56.6-129.9) | 144.3 (102.6-190.5) | 100.7 (72.9-154.9) | 146.5 (100.3-201.5) | 118.1 (83.7-153.4) | 82.8** (55.6-134.5) |
| Butyrate | 6.0 (1.1-11.9) | 10.5 (4.7-15.4) | 6.1 (3.2-10.5) | 1.7** (0.38-9.0) | 11.5 (5.8-16.3) | 5.2* (0.93-10.4) | 9.1 (3.9-13.1) | 6.9 (3.6-15.8) | 1.8* (0.39-9.2) |
| Propionate | 14.9 (3.8-29.9) | 24.6 (16.9-34.9) | 22.4 (6.7-36.7) | 4.6*** (1.3-11.4) | 22.1 (13.8-34.9) | 11.6 (3.1-29.3) | 27.9 (15.8-36.9) | 27.1 (10.3-35.0) | 6.1*** (1.4-14.5) |
| Valerate | 1.1 (0.36-2.0) | 1.9 (0.70-3.0) | 1.3 (0.52-1.9) | 0.29** (0.14-1.1) | 1.5 (0.66-3.0) | 0.95 (0.32-1.9) | 1.5 (0.63-2.7) | 1.4 (0.76-2.0) | 0.36** (0.14-1.2) |
| Isobutyrate | 0.55 (0.14-1.6) | 1.3 (0.54-1.9) | 0.50 (0.12-1.7) | 0.20*** (0.06-0.70) | 1.4 (0.69-3.1) | 0.42** (0.12-1.3) | 0.89 (0.40-1.8) | 0.64 (0.41-2.0) | 0.20** (0.07-0.63) |
| Isovalerate | 0.94 (0.31-2.2) | 1.9 (1.0-3.4) | 0.90 (0.42-2.0) | 0.41*** (0.09-1.2) | 2.2 (1.4-5.4) | 0.71** (0.23-2.0) | 1.8 (0.71-2.9) | 1.6 (0.87-3.7) | 0.45** (0.11-1.0) |
| Lactate | 3.3 (1.6-29.9) | 1.8 (1.3-2.5) | 10.6** (2.4-46.3) | 16.2** (2.4-80.8) | 2.4 (1.4-2.6) | 5.8* (1.7-39.4) | 2.4 (1.3-22.2) | 2.7 (1.5-12.6) | 10.6 (2.2-56.5) |
| Succinate | 8.2 (2.8-37.1) | 8.2 (2.1-20.1) | 23.4 (3.5-49.7) | 5.0 (2.5-37.1) | 8.3 (2.4-22.7) | 8.1 (2.8-42.2) | 4.4 (2.8-21.4) | 11.3 (3.5-35.6) | 6.5 (2.7-38.0) |

Values are presented as medians and interquartile ranges (IQR) in μmol/g feces. Comparisons by non-parametric Mann Whitney U test (2 groups ) or Kruskal Wallis test (3 groups, with Bonferroni post-test for multiple comparison) versus none or never breastfeeding category. *** p<0.001, ** p<0.01, * p<0.05. †Breastfeeding status as stool sample collection.‡Total SCFA represent the sum of acetate, butyrate, propionate, valerate, isobutyrate, and isovalerate.

Table S4. Relative proportions of total SCFA according to breastfeeding status and duration restricted to vaginally delivered infants not receiving IAP.

|  |  | **Breastfeeding status† n=76** | | | **Ever breastfed n=78** | | **Exclusive breastfeeding duration n=78** | | |
| --- | --- | --- | --- | --- | --- | --- | --- | --- | --- |
| **Metabolite % (IQR)** | **All infants (n=78)** | **None (n=24)** | **Partial (n=26)** | **Exclusive (n-26)** | **No (n=11)** | **Yes (n=67)** | **Never (n=21)** | **<3 months (n=25)** | **≥3 months (n=32)** |
| Acetate | 78.6 (72.3-89.4) | 77.6 (71.0-81.2) | 76.4 (68.1-88.0) | 86.7* (76.9-98.2) | 77.9 (75.3-81.6) | 78.7 (72.2-92.5) | 79.3 (71.1-85.1) | 76.8 (69.5-81.9) | 84.4 (75.6-95.0) |
| Butyrate | 4.0 (0.95-7.7) | 5.7 (4.3-7.5) | 3.7 (1.8-7.6) | 1.9 (0.25-8.4) | 5.9 (4.7-6.8) | 3.8 (0.81-7.7) | 4.4 (3.1-6.1) | 5.8 (2.6-8.5) | 2.0 (0.38-7.3) |
| Propionate | 11.7 (3.9-17.2) | 14.3 (10.5-17.8) | 14.6 (4.4-19.8) | 4.3** (0.94-10.3) | 13.4 (8.5-15.9) | 11.0 (3.3-18.6) | 13.4 (7.1-19.8) | 14.7 (8.2-18.8) | 4.8 (2.2-14.1) |
| Valerate | 0.72 (0.34-1.4) | 0.97 (0.62-1.5) | 0.73 (0.31-1.0) | 0.45* (0.14-1.1) | 0.83 (0.58-1.6) | 0.70 (0.31-1.3) | 0.84 (0.56-1.5) | 0.79 (0.60-1.4) | 0.47 (0.20-1.1) |
| Isobutyrate | 0.36 (0.14-0.86) | 0.80 (0.36-0.91) | 0.33 (0.15-0.69) | 0.22** (0.08-0.65) | 0.85 (0.58-1.0) | 0.30* (0.11-0.74) | 0.63 (0.21-8.6) | 0.57 (0.30-0.94) | 0.18 (0.10-0.56) |
| Isovalerate | 0.75 (0.23-1.5) | 1.1 (0.88-2.0) | 0.74 (0.22-1.5) | 0.43** (0.13-1.2) | 1.4 (1.1-2.4) | 0.61** (0.21-1.4) | 0.94 (0.25-1.4) | 1.1 (0.75-1.8) | 0.43 (0.15-1.1) |

Values are presented as median relative proportions (%) and interquartile range (IQR). Comparisons by non-parametric Mann Whitney U test (2 groups) or Kruskal Wallis test (3 groups, with Bonferroni post-test for multiple comparison) versus none or never breastfed category. *** p<0.001, ** p<0.01, * p<0.05. †Breastfeeding status at fecal sample collection.
